# Smart Cities of the Future as Cyber Physical Systems: Challenges and Enabling Technologies

**DOI:** 10.3390/s21103349

**Published:** 2021-05-12

**Authors:** Antonio Puliafito, Giuseppe Tricomi, Anastasios Zafeiropoulos, Symeon Papavassiliou

**Affiliations:** 1 Department of Engineering, University of Messina, 98100 Messina, Italy; gtricomi@unime.it; 2 Consorzio Interuniversitario Nazionale Informatica, 00185 Rome, Italy; 3 Zografou Campus, National Technical University of Athens, 15780 Athens, Greece; tzafeir@cn.ntua.gr (A.Z.); papavass@mail.ntua.gr (S.P.)

**Keywords:** cloud, IoT, smart cities, embedded systems, wireless systems, cyber physical systems, online social networks, software-defined networks

## Abstract

A smart city represents an improvement of today’s cities, both functionally and structurally, that strategically utilizes several smart factors, capitalizing on Information and Communications Technology (ICT) to increase the city’s sustainable growth and strengthen the city’s functions, while ensuring the citizens’ enhanced quality of life and health. Cities can be viewed as a microcosm of interconnected “objects” with which citizens interact daily, which represents an extremely interesting example of a cyber physical system (CPS), where the continuous monitoring of a city’s status occurs through sensors and processors applied within the real-world infrastructure. Each object in a city can be both the collector and distributor of information regarding mobility, energy consumption, air pollution as well as potentially offering cultural and tourist information. As a consequence, the cyber and real worlds are strongly linked and interdependent in a smart city. New services can be deployed when needed, and evaluation mechanisms can be set up to assess the health and success of a smart city. In particular, the objectives of creating ICT-enabled smart city environments target (but are not limited to) improved city services; optimized decision-making; the creation of smart urban infrastructures; the orchestration of cyber and physical resources; addressing challenging urban issues, such as environmental pollution, transportation management, energy usage and public health; the optimization of the use and benefits of next generation (5G and beyond) communication; the capitalization of social networks and their analysis; support for tactile internet applications; and the inspiration of urban citizens to improve their quality of life. However, the large scale deployment of cyber-physical-social systems faces a series of challenges and issues (e.g., energy efficiency requirements, architecture, protocol stack design, implementation, and security), which requires more smart sensing and computing methods as well as advanced networking and communications technologies to provide more pervasive cyber-physical-social services. In this paper, we discuss the challenges, the state-of-the-art, and the solutions to a set of currently unresolved key questions related to CPSs and smart cities.

## 1. Introduction

Cyber physical systems (CPS) are complex, heterogeneous, distributed systems where the cooperation among cyber components (e.g., sensors, actuators, and control centers) and physical processes (e.g., temperature control, traffic management, and fire detection) is deeply intertwined. A CPS is defined as a system where the computation, networking, and physical processes are integrated to monitor and control physical environments [1]. The diffusion of CPSs is strictly related to the advent of the Internet of Things (IoT), a collection of devices with limited computational capabilities that expose their services to the internet, following a TCP/IP stack [2].

Sundmaeker et al. [3] stated that IoT devices were born in the year 1999 in the MIT Auto-ID Lab as technologies that included bar codes, smart cards, sensors, voice recognition, and biometrics. In 2005, Srivastava [4] identified the trend pushing the technology toward a pervasive dimension and, in particular, pushing devices in that direction. Sundmaeker, again in [3], deeply analyzed the IoT concepts and perspectives from several points of view, providing an interesting categorization.

Available IoT devices may be equipped with a microcontroller unit (MCU) and/or a microprocessor unit (MPU) [5], exploiting their facilities to manage sensors (smoke, gas, fire, presence, cameras, and more), actuators (lights, valves, traffic lights, motors, and more) during their life-cycle; at the same time, they may run programs that pre-process the physical signals to produce data that is useful for several purposes. For example, a single smoke sensor is not sufficient to identify a fire (a cigarette could deceive it).

A traditional fire system delivers the perceived signal to a central processing system that correlates the signals with other sources and decides whether to activate the alarms, while also informing firefighters and surveillance. A CPS typically operates side by side with a multitude of other CPSs (e.g., vehicles, factories, buildings, hospital, street, and more), making it difficult, or even unfeasible, to have a unique framework to manage the resulting whole system for several reasons:

- Administrative: The environments belong to several owners, private or public, that are free to make their own choices according to various factors: financial, bureaucratic constraints, etc.
- Technologies advancement: CPS realized at different times adopts different technologies.
- Incompatibility with previously deployed technologies.

CPS federation is the new direction followed by many researchers, which allows integrated autonomous CPSs through federation to manage, coordinate, and organize sensors, actuators, and host resources and to provide support for the development and maintenance of high-level services [6].

A first example of a CPS is the Smart Building (SB), historically defined in 1981 with the term Intelligent Building, coined by the United Technology Building Systems Corporation, then, implemented in the City Place Building in Hartford, Connecticut [7]. “Smart” buildings are mostly customized control systems able to provide basic automatic management facilities of the installed devices (e.g., smoke and fire sensors, ventilation peripherals, and heating systems).

In 2009, the European Commission’s Information Society provided a long and complex definition of a Smart Building [8]; a simplified version of this is the following: a Smart Building is an integrated system based on the IoT and Ubiquitous Computing facilities that is able to take advantage of a range of computational and communication infrastructures and techniques. The Smart Building concept is easily configurable in several scenarios, modifying its characteristics to obtain various results. For example, a SB specialization is related to the industrial context, where the physical processes commonly monitored by a SB (e.g., HVAC, fire, and intrusion control systems) are added to the controls related to the production processes. This way, the system (in this case a smart factory) will be able to monitor and quickly react to emergencies coming from the security system.

Another category of CPS that is relevant and interesting is that of public infrastructures. Streets, public buildings, and undergrounds are commonly considered part of a smart city [6]. Efforts in this direction are devoted, both at the institutional level (as in the EU community) and at the academic and industrial levels as well, focusing on how to orchestrate traffic vehicles, enhance the security of the citizens, monitor air conditions, support public transportation systems, make faster and safer rescue operations, and so on.

A smart city experiences a combination of several CPSs that have to be interconnected with the others by exchanging data, raw or pre-processed, enabling workflows involving the city to support everyday citizens’ lives. With regard to the realization of such interconnections, the following question arises: Do the CPSs belonging to the same administrative domain? The answer guides us toward one of the following two solutions:

- If the answer is yes, we can model such interconnection in a tightly coupled way by adopting a Software-Defined Building approach.
- If the answer is no, we are obliged to consider a loosely coupled interaction where not all the capabilities of the systems are shareable, according to a federated cooperative approach.

**Figure 1:**
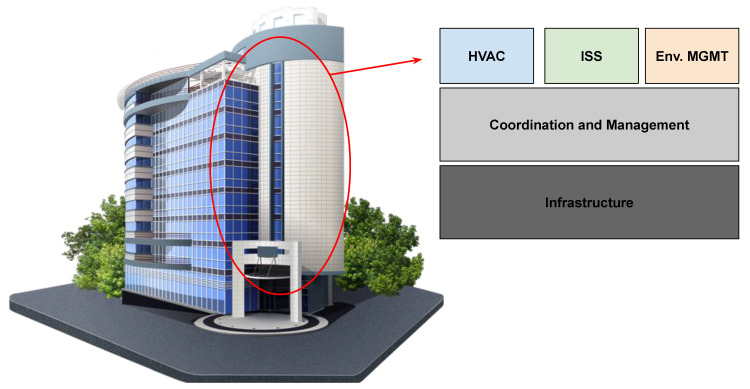
An example of coexistent cyber physical systems (CPSs) in a software-defined building.

A Software-Defined Building (see Figure 1) can be defined as a building where, in line with the Software-Defined principles, the infrastructure and its composing devices are managed in a common way, offering the upper layer the functionalities to be managed. The upper layer is the management layer, where several facilities are available:

- to control and manage devices of the lower layer,
- to orchestrate, aggregate, filter, and preprocess data coming from the infrastructure layer, and
- to offer functionalities exploitable by the applications that provide abstraction of the IoT devices available in the infrastructure layer.

**Figure 2:**
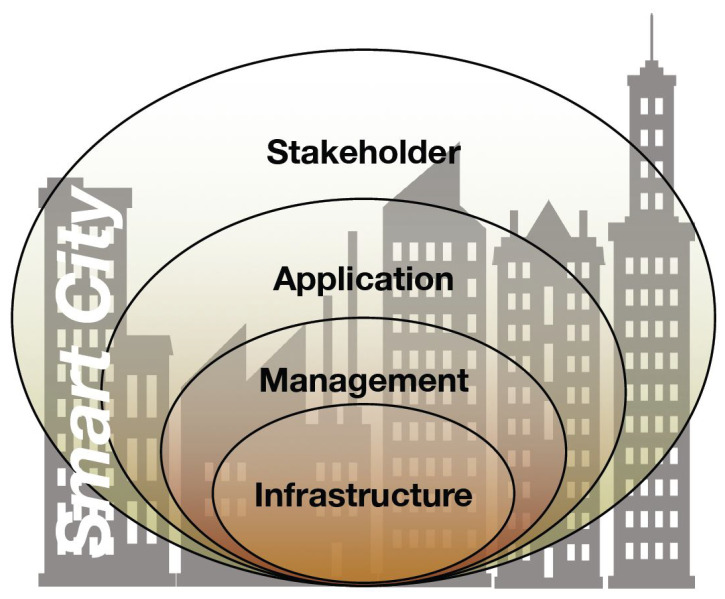
The four-layer model of a smart city.

A summary of this vision is depicted in Figure 2, representing the well known four-layer model [9]. Following a bottom-up approach, the Information and Communication Technologies (ICT) infrastructure composed of sensors and actuators represents the smart city foundation. These objects are spread in the urban area and include public and private devices, such as traffic lights, smart billboards, bus GPS, lamp posts, air pollution and weather stations, smart cameras, and citizens’ mobile devices. The infrastructure layer manages Cloud and Edge computational resources, the networking resources, and the storage and commuting facilities to collect, manage, and elaborate data.

In this way, the infrastructure becomes very complex and has to be properly managed, therefore this layer has to provide core mechanisms for the smart city, enabling as many as possible users/citizens to access all the available resources. Evolving 5G and future technologies for network and computer infrastructure management have to be considered as well. On top of the infrastructure layer is the **management** layer, which provides platform-advanced features and, at the same time, enhances the infrastructure core mechanisms.

This may include AAA, monitoring, profiling, SLA and QoS mechanisms, security, privacy, policies, orchestration strategies and placement, credit-reward systems, incentive mechanisms, and so on. The **Application layer** enables the smart city scoped services and applications to operate on several urban contexts, such as mobility, waste, public safety, energy, e-health, water, and BIM, to name a few. Finally, the **stakeholder** layer includes all the players involved in the smart city everyday life (e.g., the municipality, medical doctors, officers, enterprises, telecommunication operators, citizens, and vehicles).

The #SmartME project [10] is a crowd-funded that plans to transform Messina into a smart city. The main goal is to distribute IoT resources throughout the municipal area, thus, enabling the creation of a ubiquitous sensing and actuation infrastructure. This infrastructure becomes a virtual laboratory used by multiple stakeholders that have contributed with their own resources. They may develop applications and services for research, business, and administrative activities on top of this infrastructure.

One of the main novelties of the #SmartME project is to set up a new, crowd-sourced and shared form of a smart city where anybody, from shops and businesses to private buildings and from public administrations to citizens, can share their sensing and actuating hardware facilities to build up infrastructure. The resulting system has to be properly managed; thus, a specific framework allows the contributors to share their resources with application developers and users to ensure a simple and powerful access to all the available resources.

In this paper, the Stack4Things (S4T) [11] was adopted, and its services and functionalities were exploited to integrate CPSs and pave the way toward the smart cities of the future (see Section 6).

More specifically, the state of the art on complex CPSs is presented in Section 2, while challenges and enabling technologies are discussed in Section 3. Mobile edge computing principles are discussed in Section 4. Section 5 focuses on next generation smart IoT, considering the presented four-layer model of a smart city in Figure 2. Section 6 presents the Stack4Things framework and highlights this architectural organization and the main technical features. Uses cases are presented in Section 7. Our final remarks are summarized in Section 8.

## 2. Complex CPSs in a Glimpse

We refer to a complex CPS as a system composed of several smaller CPSs that belong to different administrative domains (such as different private owners or a mix of private and public owners), according to a federated cooperative approach. In this scenario, a crucial topic is how the shared CPSs facilities are exploited [12,13]. During the federation process, the domains involved have to sign an of agreement that defines the facilities shared and the classical Service Level Agreement (SLA) used for the cooperation [14,15,16,17].

The cooperation system has to avoid SLA’s overwhelming limitations as defined and agreed by the involved entities. For this reason, coordination and cooperation patterns for service selection were also evaluated, with consideration of the approaches adopted in the literature, both in the cases of brokered and decentralized ones.

**Figure 3:**
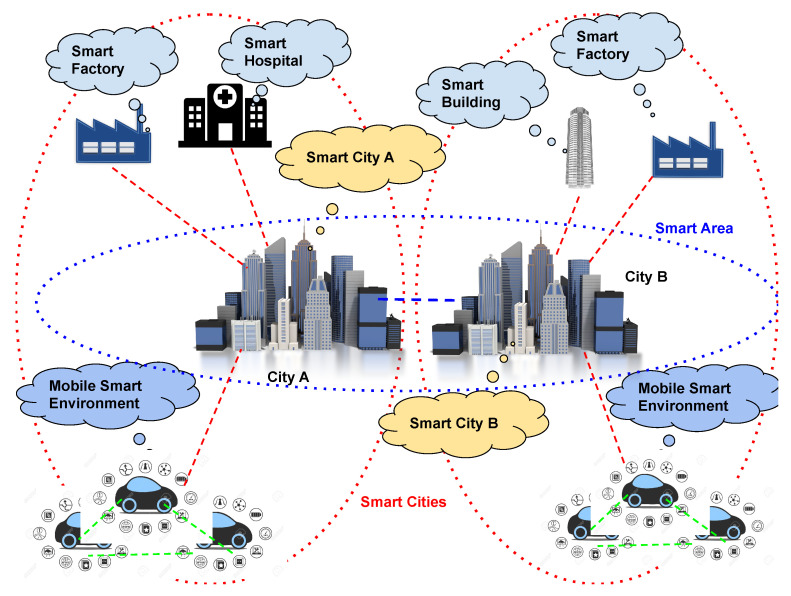
An example of a Smart Area.

In particular, the complex CPS represents the next step in the smart city research. This aggregation of CPSs, as shown in Figure 3, represents a new dimension of Smart Cities that is extensible to wider environments. As an example, it can represent a Smart Area (composed of an aggregation of Smart Cities), or a Smart Country, and so on. Federated cooperation among CPSs enables several advantages, including:

- allows more data available for applications running on CPS,
- enables the sharing of computation resources between CPSs, and
- creates an infrastructure enabling the exploitation of Cloud, Fog, Edge, and Cloud Continuum approaches without increasing the cost for the CPS owner.

All the advantages discussed until now regarding the cooperation among CPSs makes possible the realization of platforms and applications, improving citizens’ lives decisively. The applications exploitable in similar scenarios are uncountable. They span from advanced traffic monitoring, management and driving utilities, to the realization of enhanced Intrusion Surveillance System based on neighborhood surveillance systems and to cooperative emergency management that supports rescue activities.

Another interesting aspect related to CPS and, in particular to CPSs cooperation, is the distribution of computation among the available computing elements. Let us make an analogy among a human and a CPS: we can assume that the eyes and the hands of a CPS are represented by the IoTs, while the body and the brain are equivalent to the Cloud. In this way, the CPS becomes a perfect infrastructure where it is possible to apply the Cloud Continuum (the paradigms in which the computation is distributed on the whole CPS exploiting Cloud, Fog, and Edge computing facilities) principles [18]; in particular, we must refer to Fog/Edge and Cloud computing technologies to complete the analogy mentioned above.

Fog and Edge computing [19,20] are paradigms of computing that operate near the periphery of a system. Indeed, they differ for where the computation occurs. The latter makes its elaboration into or near the Edge devices (commonly IoTs, but it is often exploited on gateways, or similar). Instead, the former moves the computation to processors connected in the same LAN or into the LAN hardware itself.

These techniques, supported by new emergent computing paradigms [21], as they are serverless, enable the CPS to be easily exploited by the applications previously discussed. In this sense, we made some preliminary studies [22] with some prototypes offering an idea of how the application of the serverless technique simplifies the setup and the re-configuration of the IoT devices through simple function calls.

In this research path, cooperation schemes and techniques for the selection of resource providers are important issues to be addressed. A literature review to understand the differences between brokered and decentralized Federated Cloud Service Providers is available in [23].

According to this vision, it is possible to consider a smart city as an ecosystem of services and relative infrastructures implementing the characteristics already discussed. In this way, the scenario depicted has to be observed through a holistic view, i.e., an all-encompassing approach combining heterogeneous services and technologies to provide a wider (or even global) solution to (Smart) city problems.

In this regard, it is necessary to realize a scalable architecture aiming at multiplexing, sharing, and reusing services and technologies on the urban scale. The goal is the creation of a homogeneous ecosystem that enables the applications to be scaled out to a metropolitan (or even wider) scope; this requires an ICT infrastructure that is open, shared, able to access storage resources, process data, provide networking, and finally sense and act on the real world.

Stack4Things [11] is a management framework allowing the user to enroll and manage resources altogether as a whole. S4T also provides customization facilities and fruition modalities for resource exploitation following the Cloud provisioning model and, specifically, a federated cooperative approach, thus, resulting in a complex CPS. By involving several stakeholders, multiple services exploiting this crowd-sourced smart city have been developed. The #SmartME experience was then extended to other italian cities, such as Turin, Padoa, Lecce, and Syracuse, through the Too(L)Smart project, thus, establishing an interesting and successful example of technology reuse and best practice adoption [24].

## 3. IoT Technologies in Smart Cities of the Future

Smart cities are continuously embracing the IoT technological evolution, combined with relevant advances in the areas of 5G networks and Mobile Edge Computing (MEC). Under this perspective, smart cities of the future can be considered as a microcosms of interconnected (physical and virtual) “objects” where advanced and human-centric services can be provided to citizens in the form of cyber physical systems.

This evolution moves in parallel with the increase in the heterogeneity of IoT technologies [25,26] in terms of the production of different types of intelligent IoT devices, the support of various communication protocols, the release of IoT platforms tackling deployment in various parts of the available infrastructure (e.g., edge and cloud), the tackling of diverse requirements stemming from various use cases and the conceptualization of various information models for semantically representing entities in a smart city.

As the number of IoT devices and solutions expands, any proprietary approach does not scale and, thus, slows the growth of the IoT ecosystem. Such heterogeneity and multiplicity make inherent the need for the design of architectural approaches that are able to support a high level of convergence and integration among existing and evolving IoT technologies. To do so, as stated at the World Economic Forum report for “Realizing the Internet of Things” [27], the arisen unique and impending IoT ecosystem challenges must be addressed not in an ad hoc and piecemeal manner, but with a holistic view.

Toward the design of next generation CPS systems for Smart Cities, novel schemes msut be developed that are able to tackle convergence, openness, and interoperability. Next-generation CPS will enable and will be enabled by current and future advances in several emerging technologies, such as 5G, cloud/edge-native computing, tactile internet, and artificial intelligence (AI), presenting a tremendous potential for the development of solutions applicable to Smart Cities.

### 3.1. Challenges and Enabling Technologies

In the following, we highlight a set of identified challenges toward the development of innovative solutions for the smart cities of the future, as well as a set of enabling technologies that can boost their development. An overview of the set of identified challenges and the technologies that can be exploited to address them is provided in Table 1.

#### 3.1.1. Need for Convergence of IoT Technologies

The mirroring of IoT objects can be a catalyst for supporting interoperability with IoT devices that support different communication protocols or semantic representations, as well as for significantly enhancing privacy and security aspects by protecting the IoT device through its digital twin (DT) in the edge part of the infrastructure [28]. This perfectly matches with the idea proposed by the *I/OCloud* concept exploiting the Stack4Things framework and presented in [29]. This can also enable IoT on a larger scale by using smaller and cheaper IoT devices that are able to perform sensing and basic computational functionalities, while pushing the heavier computational functionalities at the edge or cloud part of the infrastructure.

Through the interpretation of different types of semantics, IoT devices and CPS systems can be managed in a neutral way in terms of semantic representation, while the collected information can be automatically interpretable and integrable by the edge and cloud part of a smart city application, avoiding lock-in in a specific modeling approach. With regard to the semantic interoperability, various information models targeted to the development of CPS have been made available, such as the W3C Web of Things [30], OMA IPSO Smart Objects [31], FIWARE NGSI-LD [32], and Web5G [33] specifications. Upon the usage of such models, efficient and secure data sharing based on distributed data management and data monetization schemes can be realized (e.g., through blockchain-based techniques), unleashing the potential for IoT data exploitation by various stakeholders [34].

#### 3.1.2. IoT Application Development and Management Taking Advantage of 5G/Edge Computing Infrastructure

Moving one step further, modularity, openness, and interoperability should be supported by design toward the development of distributed and self-adaptive IoT applications. The adoption of cloud-native principles and the adherence to microservices-based architecture along with lightweight containerization mechanisms based on containers, allows the flexible, scalable, and dynamic composition of IoT applications with different application components. Each IoT application component can be independently deployable and orchestratable in the edge or cloud part of the infrastructure.

Generic IoT functions or enablers can be easily introduced and constitute a part of the overall application—a characteristic that cannot be feasible in a monolithic architectural approach. As discussed in Section 6.2, through the exploitation of cloud and edge computing technologies, distinct IoT functions (e.g., IoT gateway, IoT distributed storage, contractual agreements, privacy, security, and distributed AI) can be executed on demand at different locations of the infrastructure and cover strict Quality of Service (QoS), data management or security requirements.

#### 3.1.3. Intelligence and Automation in Edge and Cloud Computing through Machine Learning (ML) Techniques

To take advantage of the aforementioned technologies and fully support interoperability aspects, the development of end-to-end semi-automated and dynamic orchestration mechanisms for IoT applications is required. Emerging cloud and edge computing orchestration solutions by jointly exploiting 5G and AI technologies can act as a catalyst for the development of novel IoT orchestration mechanisms. 5G and IoT worlds evolution are highly interrelated, as 5G is considered an enabler for the support of massive IoT (MIoT) interconnecting numerous devices that require ubiquitous connectivity, whether mobile, nomadic, or stationary, as well as more advanced solutions that may be categorized as Critical IoT with advanced security, automation, and reliability requirements.

To cover the needs of numerous IoT applications with diverse requirements, the IoT slice concept has been introduced following similar network slicing specifications in the 5G domain. An IoT slice regards a partition of the available network and the programmable infrastructure that can be reserved and isolated for serving IoT application needs.

Context awareness is important for mapping application needs to IoT slice specifications and assuring high QoS and Quality of Experience (QoE) levels. Over a dedicated context-aware IoT slice for an IoT application, semi-automated orchestration mechanisms can be applied, deploying and managing the operation of the application components and IoT functions in both edge and cloud computing.

Serverless techniques, in particular FaaS approaches, can be easily exploited to create the above-mentioned IoT slices. As described in Section 7.2, this is possible because FaaS applied on IoTs provides dynamic mechanisms to modify the behavior of IoTs that are easily exploitable by the orchestration part. AI along with formal control-theoretic tools can introduce automation in the orchestration operations, especially at the edge of the network, by injecting intelligence (even in the form of plugins or functions as described respectively in Section 6 and Section 6.2) at the edge and supporting time-sensitive distributed decision making [35,36].

#### 3.1.4. Interaction and Feedback on Behalf of the End Users through Easy-to-Adopt Human-Centric Interfaces

Next generation CPS interconnection and management solutions that facilitate the smart city of the future operation have to be human-centric, serve human needs, and lead to applications that increase social well-being. Tactile and haptic communications (e.g., the real-time transmission of haptic information, such as touch, actuation, motion, vibration, and surface texture) based on physical or remote interactions have to be supported through easy-to-use human-machine interfaces, while low latency and high availability, reliability, and security requirements also have to be guaranteed [37,38].

The convergence of tactile internet technologies with IoT slicing and IoT object mirroring technologies appears as promising for tackling these needs. The term “tactile internet” was defined by the IEEE P1918.1 [39] as: “A network or network of networks for remotely accessing, perceiving, manipulating or controlling real or virtual objects or processes in perceived real time by humans or machines”.

Involving human-in-the-loop (HITL) by considering human perception and enhancing the collaboration and interaction between humans and machines in real, virtual, and remote environments based on explainable AI mechanisms, is crucial. Augmented and virtual reality (AR/VR) technologies and intelligent IoT devices can undertake a significant role in this process. The potential delivery of physical tactile experiences, remotely or locally, enables the delivery of skills in digital form. This novel domain, named the Internet of Skills (IoS) [40], will revolutionize the way we generally interact with our surroundings, creating a new perspective for the smart cities of the future, based on the evolving concept of CPS.

Of significant importance is the consideration of more realistic user behaviors in accessing the available communications and computing resources, thus, giving rise to risk-aware and cognitive data offloading approaches in MEC environments and, thereby, realizing an HITL methodology.

#### 3.1.5. Efficient IoT Data Storage, Representation, and Management

Data management is key to a viable IoT strategy. The nature of IoT data is frequently heterogeneous and unstructured when collected at the cloud. Data lakes have become popular for storing, crawling, cataloging, and indexing data from IoT devices (e.g., Delta Lake [41]). One step further is the converting of data lakes to knowledge graphs [42]. Knowledge graphs include the power of semantics, making possible intelligent analysis of the data and further data monetization.

Based on the collected data, IoT devices also need to be able to learn from each other, which means that there is a need for leveraging on all the local AI models created on IoT/edge devices. Federated learning techniques make this possible since the IoT end devices use their local data to train the machine learning models required by central services. IoT devices send the model updates rather than raw data to the centralized services for aggregation [43]. Federated learning has proven to help in terms of the data privacy and accuracy of machine learning solutions.

More generally, this is valid for all the distributed learning programming techniques that may take advantages from the IoT facilities exploitation, as made in [44] with the support of the framework presented in Section 6. Typically, the data streaming from a few connected devices may be manageable; however, additional storage and privacy needs can become an issue as more devices come online. A blockchain-based design for the IoT makes possible a distributed access control and data management by skipping a centralized trusted authority and instead empowering the users with data ownership.

## 4. Mobile Edge Computing for the Internet of Things

In this section, we present the main aspects that are examined considering the interplay between cloud and edge computing resources toward the development and management of IoT applications in a smart city. In most IoT applications, diverse computer, network, and storage requirements have to be satisfied to provide the required levels of Quality of Service (QoS) and Quality of Experience (QoE) for the end users.

Such requirements have to be satisfied while taking into account a set of constraints that may be related to the existence of limited resources at the edge part of the infrastructure, the dynamic network topologies based on the activation and deactivation of IoT nodes and the fluctuations in the posed workload by the end users.

The trade-off between the optimal usage of the available resources and the associated impact on the IoT applications performance has to be dynamically assessed and must lead to resource management actions at the edge and the core part of the infrastructure. To achieve this, part of the IoT functions that compose an IoT application graph may be executed at the edge part of the infrastructure, while another part may be executed at the cloud part. Decision making regarding the amount of resources to be allocated per part of the infrastructure (edge, cloud computing part or access, transport, and core network part) is usually made by centralized orchestration mechanisms that have a full and accurate view of the current status of both the available resources and the application performance.

Various orchestration approaches have been developed in recent years under work realized within different working groups in the areas of Multi access Edge Computing (MEC), Network Function Virtualization (NFV) and—under a wider umbrella—5G technologies. One of the three main categories of 5G usage scenarios regards the Massive Machine Type Communications (mMTC) where very dense IoT deployments can take place, particularly in smart city environments. In the MEC approach, focus is given to the application part that is placed at the edge of the infrastructure.

Use cases include applications offering video analytics, location-based services, augmented reality, optimized local content distribution, and data caching at the edge. MEC applications may be provided by application providers or telecom operators. In the latter case, a MEC application is part of a service offered by the telecom operator. In the NFV approach, focus is given to satisfying the network requirements posed by the application through the deployment of network services composed of a set of virtual network functions. Such network services are provided on behalf of telecom operators. Use cases include virtual firewalls, IP multimedia subsystem controllers, virtual Customer Premises Equipment (CPE), virtual authentication, authorization and accounting functions, and virtual content delivery networks (CDN).

However, moving toward the next generation smart IoT applications, there is a need for convergence of MEC and NFV technologies and the support of the end-to-end management of IoT applications considering both application-oriented and network-oriented performance metrics. Radical new applications can be developed through the joint adoption of these technologies. Several 5G research activities are taking place toward this direction taking advantage of the evolution of 5G orchestration mechanisms and dynamic network slice management approaches [45].

5G was designed to leverage network software technologies, such as NFV and MEC, to expose customized network instances and resources at the edge of the infrastructure to vertical stakeholders [45]. One approach proposed within the ETSI MEC ISG considers that edge computing applications can be considered as VNFs and embedded in 5G orchestration ecosystems [46]. In this case, the edge computing VNF is composed of multiple application components (each one hosted within a VM or a container). Another approach considers independent orchestration ecosystems for the network services and the edge computing application, with separation of the concerns between the two orchestration loops [45].

In this case, an IoT-oriented network slice is managed by a telecom operator and made available to application providers. Over the IoT-oriented network slice, the application providers are able to deploy and manage the IoT applications, taking advantage of the evolving 5G capabilities.

In addition to approaches for the convergence of MEC and NFV technologies, various application orchestrators targeted to edge computing environments were also made available, stemming from both open-source communities and commercial releases (e.g., KubeEdge framework, FogFlow IoT edge computing framework, and K3S lightweight Kubernetes framework).

These orchestrators include modules for managing application components deployed at the cloud and edge part of the infrastructure, as well as managing the IoT devices interconnected at the edge components. Specific resource managers are made available for managing the deployment of—part of or entire—IoT applications at the edge and the provision of continuous feedback regarding the status of the edge application components (e.g., health checks, failures, and self-healing reporting).

## 5. A Holistic Approach toward Next Generation Smart Cities Application Management

In this section, we present an end-to-end approach for tackling the lifecycle management of the design, development, and orchestration of IoT-based applications over cloud and edge computing resources, targeted to smart city environments. We tackle issues related to the design of IoT applications based on the adoption of cloud-native principles and the development and adoption of generic IoT functions that can serve generic application requirements, and we cover needs that are present in various application domains, the orchestration of IoT applications over dynamic programmable infrastructure, and the appropriate modeling of IoT devices to tackle interoperability issues.

The proposed approach is in accordance with the four-layer model of a smart city that is presented in Figure 2. In the Infrastructure layer, we consider the management of a programmable network, computer, and storage infrastructure in the various parts of the network, including the management of resource-constrained devices at the edge part. In the Management layer, we consider the development of intelligent orchestration mechanisms, able to tackle the lifecycle of IoT application deployment and runtime management. In the Applications layer, we consider a set of techniques and best practices for developing distributed IoT applications.

The proposed approach aims to tackle the set of challenges that are identified in Table 1. Convergence aspects of IoT technologies is mainly tackled in the IoT Applications and the Infrastructure Management layer, where the design of generic IoT enablers and Digital Twins can be used to provide interoperable functions that can be embedded by design or on demand in the provided IoT applications. Development of efficient and scalable IoT applications is also tackled in the Applications layer based on the adoption of cloud-native principles and the exploitation of evolving containerization technologies.

Improved intelligence and automation is supported by IoT applications management mechanisms that are provided by emerging orchestration platforms for cloud and edge computing resources. Efficient IoT data storage is partially tackled in the Infrastructure management part with the provision of storage capabilities at different parts of the infrastructure, as well as the IoT applications development part with the provision of various generic functions that support distributed data management and analysis techniques. Finally, the design of human-centric IoT solutions is also tackled in the Infrastructure management part where the IoT devices can provide advanced interfaces for human-machine interactions.

### 5.1. IoT Applications Development

An IoT application is represented in the form of an application graph, following cloud-native principles and enabling the enforcement of a cloud-native IoT management approach. The application graph consists of a set of components. Each component is considered as a microservice that exposes a set of APIs. It is usually packaged in the form of a container or a Virtual Machine (VM), while its lifecycle is managed by an agent.

It is assumed that each application component is independently manageable, while it may have a lightweight and (where required) short lifetime, which is key for supporting scalability and reliability characteristics. Smooth integration between edge and cloud computing mechanisms can be realized to optimize end-to-end IoT applications delivery, since application components may be deployed and managed at the edge or cloud part of the infrastructure, considering a continuous interplay and interaction among them.

Part of an application graph can be a generic IoT function that serves specific application needs. The portability of such functions in the edge and the cloud part of the infrastructure is considered a catalyst for enabling mass, scalable, and efficient development and deployment of cloud-native IoT solutions. Generic IoT functions can support IoT-oriented functionalities (e.g., distributed data management, data aggregation, security, authentication, and failure handling), as well as functionalities at the edge of the infrastructure (e.g., service discovery, distributed AI, and telemetry).

Such functionalities are considered as assistive functionalities for the provision of IoT applications and may be provided following a service mesh approach. A service mesh enables managed, observable, and secure communication across a number of microservices (which formulate an IoT application graph), making it easier for IoT application developers and providers to focus on creating and managing applications for their users, while being able to adopt and reuse a wide range of generic IoT functionalities.

### 5.2. IoT Applications Management

IoT application management may take place based on an end-to-end orchestration platform able to manage the deployment and management of IoT workloads over the available edge and cloud computing infrastructure, considering the set of requirements per IoT application and the interplay between cloud and edge resources. A set of existing open-source orchestration frameworks can be considered (e.g., KubeEdge, and K3S). We will refer to the main modules and functionalities that have to be supported. These modules concern the IoT applications manager, the cloud/edge resource managers, and the IoT device managers.

The IoT applications manager has the main responsibility for tackling the operational lifecycle of cloud-native IoT-containerized applications. This includes modules for managing application components deployed at the cloud and edge part of the infrastructure considering the interplay between cloud and edge resources in terms of the resource usage efficiency and performance aspects, as well as managing the IoT devices interconnected at the edge components. The IoT applications manager has a view on the allocated resources for serving each application graph.

This produces a deployment plan that is realized over the materialized network slice in case of a deployment over 5G infrastructure or over the available compute clusters in the case of a pure cloud/edge computing infrastructure. The deployment plan guides the instantiation of the containerized application components at the cloud and edge computing part of the infrastructure. The IoT applications manager interacts with the resource manager at the cloud and edge part of the infrastructure.

The resource managers are responsible for managing the deployment part at the edge/cloud part of the infrastructure, the provision of continuous feedback regarding the status of the edge/cloud application components (e.g., health checks, failures, and self-healing reporting), and the related consumption of resources. Each resource manager is able to handle events related to computer offloading and mobility aspects, taking advantage of the activated IoT functions. They are able to manage various edge/cloud clusters, considering the deployment needs of each application and the available VIMs and/or computing clusters.

IoT device managers are also provided for synchronizing the device updates from the cloud to the edge node and vice versa and scheduling actions on the IoT device, considering the supported APIs and the exposed functionalities per intelligent IoT device. Through a continuous interaction among the main application manager, the various resource managers and the IoT device managers, end-to-end orchestration of IoT applications over programmable infrastructure is taking place.

### 5.3. Infrastructure Management

Infrastructure management regards both the management of the network and computational resources that are required for the provision of the various IoT application parts, as well as the management of the deployed IoT devices at the edge part of the infrastructure. We provide details per category regarding resource management.

Network, computer, and storage resource management has to be provided based on the needs of each IoT application to guarantee the optimal usage of the available resources, while providing the required QoS levels. In the case of 5G environments, by considering the set of IoT applications requirements, a context-aware IoT slice has to be created and managed. Each IoT slice is going to be instantiated, given the request for the deployment of an IoT application.

The main types of the considered slices regard IoT services are classified as massive Machine Type Communication (mMTC) services and URLLC services. mMTC relates to a very large number of devices transmitting a relatively low volume of non-delay-sensitive data (leading to the creation of a Massive IoT Slice), and URLLC relates to services with stringent requirements for capabilities, such as throughput, latency, and availability. The IoT network slice is realized and managed on behalf of a telecom operator through the deployed orchestration (e.g., the Network Function Virtualization Orchestrator—NFVO) components and network management systems.

Following evolving 3GPP specifications, the mechanisms that manage the IoT application netwwork slice are responsible for realizing the setup of virtual (isolated/shared) 5G networks built upon the combination of standard Network Functions, such as the User-Plane Function (UPF), the Session Management Function (SMF), and the Network Exposure Function (NEF), etc.

Cloud and edge computing resource management concerns the reservation of the required computational and storage resources at the cloud and edge part of the infrastructure. Multiple edge locations and edge clusters are considered, with the continuous interplay of resource management mechanisms between cloud and edge resources as well as among edge resources.

Interfaces toward Virtual Infrastructure Managers (VIMs) and computer resource clusters allow the reservation of resources and to create tenant spaces for hosting IoT application components and VNFs at edge computing facilities. Computer offloading mechanisms can be supported for deploying workloads closer to users and IoT devices, and properly scheduling the execution of resource-intensive tasks.

Management of IoT devices can be realized in two ways; through management of their virtual counterpart (digital twin) or through management mechanisms applied by IoT agents in the IoT devices. In the first case, the developed software components are considered as an extension of the IoT application graph and can be managed by the developed orchestration mechanisms, while, in the latter case, interfaces for the management of IoT devices are made available.

In both cases, proper abstractions for the supporting management of the IoT devices represented based on different semantic models have to be provided. Focus has to be given to techniques that enable semantic interoperability even if different information models are used. To achieve this, alignment with well-defined information models (e.g., the FIWARE data model and W3C Web of Things) has to take place, considering the IoT node capabilities, communication protocols, and type of the sensed context.

Adoption of the provided solutions by humans, through the support of interactiosn with real and virtual devices, exploiting advancements in AR/VR technologies, is considered as crucial. Given that tactile interaction refers to a level of responsiveness that works at a human scale, the combination of efficient deployment and provision of IoT applications with effective, usually synchronous, and human-friendly interaction among humans and IoT devices has to be supported.

Mechanisms for delivering a seamless user experience also have to be considered. Analysis of the way that people interact with the IoT devices can be realized, leading to mechanisms that learn from their behavior and adapt the context provisions accordingly.

## 6. Stack4Things as the CPS Framework

Stack4Things (S4T) is a platform that extends the OpenStack framework with IoT capabilities. The design of S4T is split into two subsystems: the first is hosted in a datacenter where IoTronic is deployed; the second subsystem is represented by a number of geo-distributed IoT devices that host the S4T device-side agents, named Lightning-Rod (LR).

**Figure 4:**
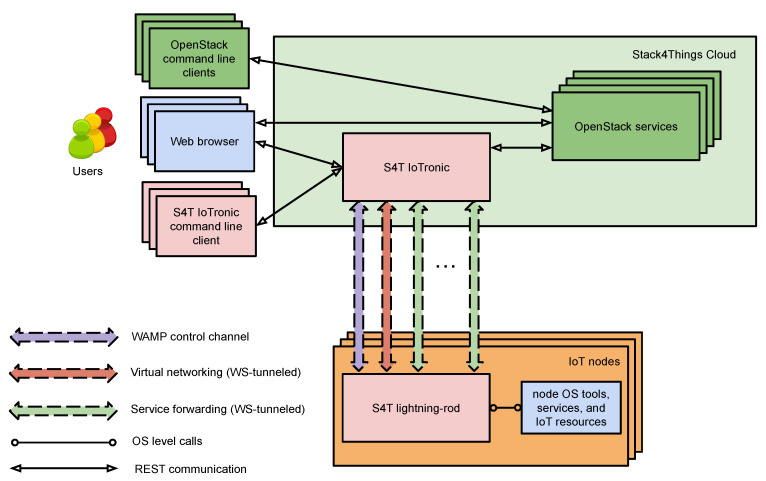
S4T architecture overview.

The communications between the Cloud-side, IoTronic, and its device-side counterpart, LR, are built exploiting a mechanism based on WebSockets with a reverse tunneling approach that is able to bypass firewall and NAT systems (see Figure 4).

As S4T is compatible with OpenStack, the interaction with other (OpenStack) services (e.g., Keystone for access management and Neutron for networks) is easily provided, and advanced user-facing features, such as containerized applications at the network edge and virtual networking are granted. In a nutshell, S4T provides the support of (among others):

1. **Authorization/ Authentication:** S4T can manage users’ authentication exploiting the OpenStack identity service called Keystone; it is also able to provide authorization to access and manage remote IoT devices.
2. **Remote access and management:**
  Exploiting the service forwarding facilities through the Cloud offered by S4T, a user can access (e.g., through vnc or ssh) their IoT devices without having to consider the device localization or networking configurations. This is possible thanks to a reverse-tunneling mechanism, based on Websockets.
3. **Remote customization/contextualization:**
  Using S4T, the application logic to be executed on the devices can be defined by an user and then distributed in form of functions and deployed on IoT devices, according to authorization and privacy policies, even at runtime. Python and Node.js are available as runtime environments in S4T.

S4T was developed keeping in mind the holistic approach introduced in the previous section. S4T strongly exploits the concepts of interoperability and network and device management through the adoption of virtualization. S4T also simplifies the development and management of applications by adopting a serverless paradigm for the network edge using the OpenStack FaaS subsystem Qinling, as described as follows.

### 6.1. S4T Virtual Networking

Stack4Things is used to create virtual networks (i.e., overlays) among distributed IoT devices. Therefore, they can reach each other as if they were on the same physical network (i.e., LAN), thus, providing a mechanism to enable VNFs on the tenant space, as discussed in Section 5.3. To enable this capability, we integrated Neutron, the networking subsystem in OpenStack, with IoTronic Therefore, we extended the Neutron capabilities to provide networking services for instances (i.e., IoT devices) deployed outside the cloud (the standard Neutron enables networking services for cloud-based instances only).

In our approach, we consider as binding-hosts (where the Neutron L2 agents are running in addition to software switches) nodes hosting the S4T WS tunnel agents while the instances are the remote IoT nodes. Consequently, Neutron ports are created and managed on these nodes (i.e, S4T WS tunnel agents hosts) along with their networking facilities (i.e., software switches). In our design, the ports are created on the cloud-side (i.e, WS tunnel agent hosts); yet, they will be attached to our approach instances, which are the remote IoT nodes located at the edge of the network, where Virtual InterFaces (VIFs) are instantiated.

**Figure 5:**
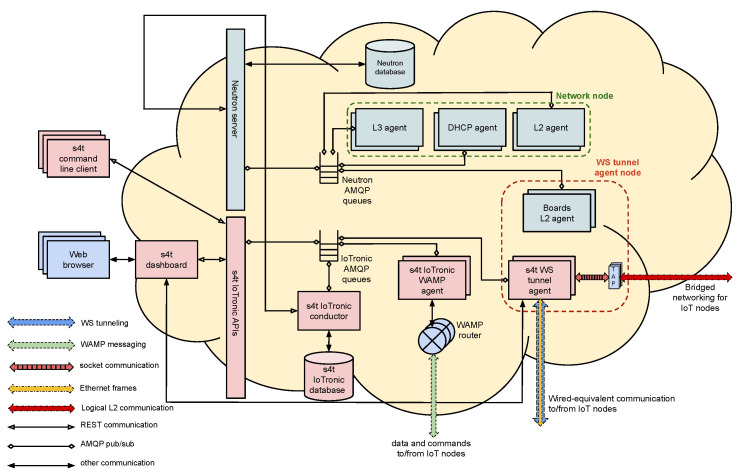
The cloud-side S4T virtual networking subsystem.

**Figure 6:**
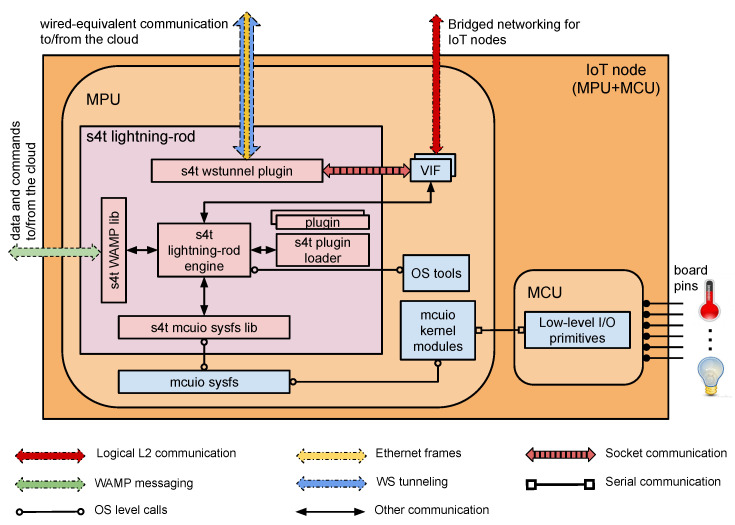
The device-side S4T virtual networking subsystem.

The S4T Cloud side networking system is illustrated in Figure 5, while the node-side architecture is highlighted in Figure 6 The proposed S4T design has been thought out considering the typical constraints of IoT environments, thereby, making the approach versatile and scalable. On the one hand, the edge nodes are completely not involved in most of the network virtualization duties since they are completely unaware of the Neutron involvement, thus, making the overall footprint of the solution inherently lightweight for them. On the other hand, since L2 agents and switching platforms are running on the cloud, the approach provides availability for mission-critical Neutron services and scalability for particularly hefty configuration requirements.

### 6.2. S4T Edge FaaS System

**Figure 7:**
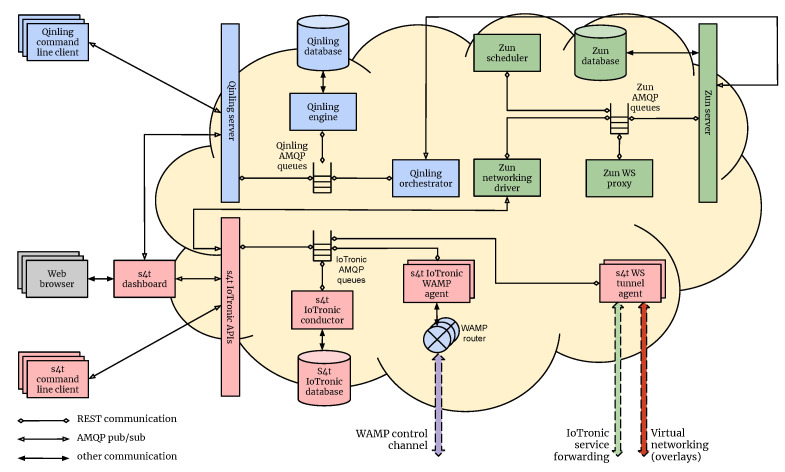
The S4T FaaS system (cloud-side).

**Figure 8:**
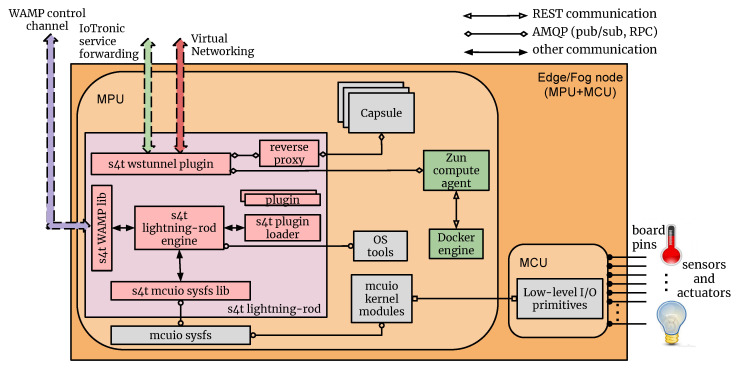
S4T FaaS system (board-side).

According to Section 5.2, the S4T framework was modeled extending the serverless (i.e., Function-as-a-Service: FaaS) paradigm to the network Edge using the OpenStack FaaS subsystem Qinling. In particular, in order to deploy functions at the edge on top of IoT devices, Qinling uses IoTronic as the networking driver for the containers (created by Zun). The architecture of the system is highlighted in Figure 7 and Figure 8. A user, in order to deploy a runtime/function on a particular IoT device, interacts, through the dashboard or CLI, with the Qinling-API server that forwards the request to the Qinling orchestrator.

This later component cooperates with the Zun-scheduler to identify the IoT device where the runtime/function should be deployed then, the Zun-API server sends a request to create, on this device, the containers needed (i.e., the capsule). To make users able to reach the capsule and in particular, the runtime container, IoTronic exposes it, on the cloud side, using a public IP address and a port, and then a WS tunnel is created between the Cloud and the IoT device. Hence, a request that reaches the cloud on that IP address/port will be forwarded to the WS tunnel and reach the device. On the device-side, the request is received through the S4T wstunnel plugin and forwarded to the reverse proxy that routes it to the correct runtime.

### 6.3. S4T Secure Web Services

**Figure 9:**
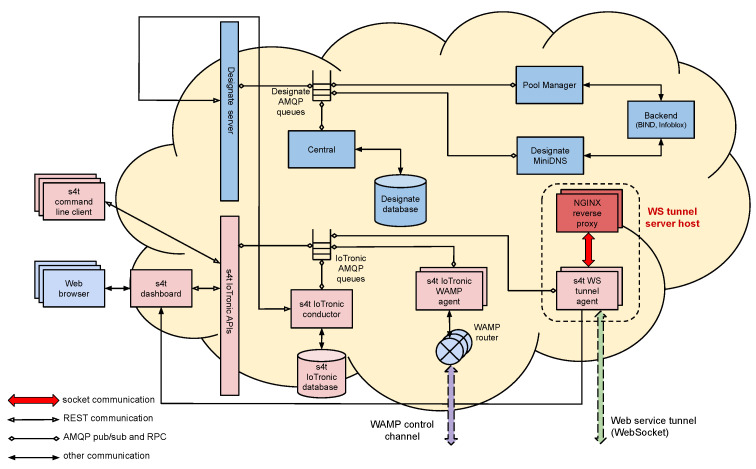
The S4T cloud-side web services system.

Our goal is to create a homogeneous environment where IoT objects interact with each other and with other components from the existing web world, offering their functionalities (e.g., sensed data) through RESTful APIs. In such a way, a device with an embedded temperature sensor can provide real-time sensed data as a web service. Smart objects can offer their web services to other devices, web services, and applications to provide appealing applications. Through S4T it is possible to expose services running on IoT devices to the web, by integrating IoTronic with the Designate—the DNS-as-a-Service system of OpenStack, as shown in Figure 9).

**Figure 10:**
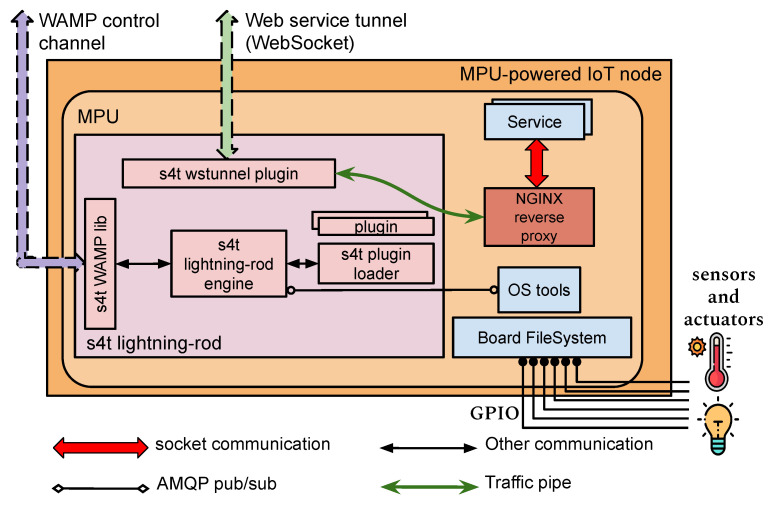
The device-side S4T web services system.

This subsystem manages the records regarding the URLs associated with the services running on the IoT devices while IoTronic deals with their reachability (i.e., request routing) by creating Websockets tunnels and configuring NGINX reverse proxies for traffic redirection/forwarding. To enable secure communication (using HTTPS) between the services and clients, S4T integrates, within the system, an automated approach (i.e., without any human interaction) based on the ACME protocol for X.509 certificate issuance and validation. This approach uses the Certbot agent (See Figure 10) with the Let’s encrypt Certification Authority (CA).

## 7. Use Cases

This section aims to synthetically describe the main experiences of CPSs to pave the way toward the *smart city of the Future*, i.e., an integrated environment with several subsystems to be integrated, controlled, and managed. We intends to demonstrate how the theoretical concepts described so far can be put into place in specific use cases exploiting the main features of the Stack4Things framework.

The use cases represent the milestones of the journey we have made to exploit the mechanisms and procedures introduced in Section 6. The first step of this journey is to create an easy-to-reuse smart city template to morph a city into a smart city highlighting the following three main concepts:

1. A CPS agnostic framework able to manage IoT devices.
2. A common point to archive data related to the CPS.
3. An optional visual system to present CPS related data.

In the second step, we want to foster the utilization of the *Computing Continuum* principles to optimize the use of CPS devices, and limit the network latency for data migration from the edge to the cloud.

In the third step, the cooperation among CPSs is exploited to show how the interaction among CPSs is beneficial to the whole system. Usually, the different CPSs of a smart city are independent subsystems that do not interact each other, while data exchange and access to sensors and actuators of other CPSs can strongly reduce execution times and simplify the management procedures (e.g., a vehicle cannot perceive a traffic light status if cooperation with the CPS controlling the traffic lights is not allowed).

### 7.1. A Skeleton for a Smart City Enabled by Cooperating CPSs

The framework presented in Section 6 is intended to realize a modular system that is able to interact with generic IoT devices; in this way, it may be easily adopted by various typologies of CPSs. To verify the validity of the solution, it was applied in real city environments as part of the #SmartME [10] initiative, where the S4T framework was used to manage environmental stations distributed in an urban area. This experience was identified and considered as a “good practice” by the Italian Ministry and further exploited in a project called *Toolsmart* [24], in which the framework was configured into a template to make its adoption in other cities easier.

**Figure 11:**
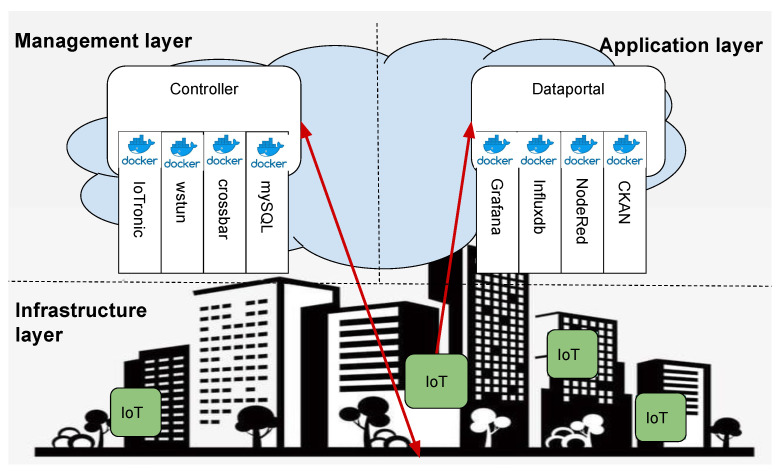
Easy to replicate architectural template of a smart city released in an Italian project called ToolSmart.

The template was configured in the form of a skeleton composed of three parts: the *Infrastructure*, *Management*, and *Presentation* layers. Figure 11 shows the architecture of the template. At the base of this template, there is the Infrastructure Layer where the Edge devices (IoT-based) are located, enabling the interaction with physical components of a CPS. These devices are managed by the Management Layer that, thanks to the S4T facilities, is able to manage their life-cycles in an agnostic way.

Indeed, the Controller entity has to set up the networking facilities enabling the edge to be monitored and controlled by the CPS’s administrator, thus, allowing differentiation of the device behavior on the basis of the plugin that is injected and executed. Due to the plugin’s logic running on the IoTs, the perceived data are sent to the Application layer (the Open data repository) where they are cataloged and pulled by the application logic defined through a graphical development platform, such as NodeRED.

**Figure 12:**
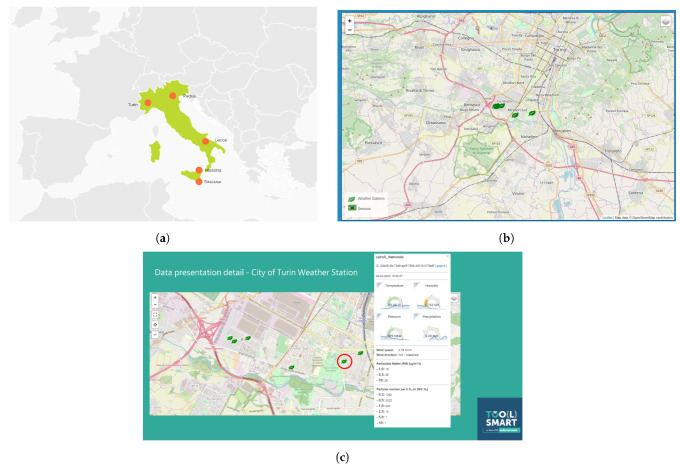
Some screenshots from the ToolSmart project: (**a**) Italian cities where the solution is applied. (**b**) Location of the Toolsmart IoT devices in Turin. (**c**) Interactive map exposed by the dataportal component.

The realization of this *smart city skeleton* is a fundamental step to enable the cities in their transformation into a “*Smart City of the Future*”. Indeed, thanks to the intrinsic characteristics (such as extensibility, interoperability, self-consistency, and replicability), the template developed allows a reduction in the amount of work to create and manage the CPS and to deal with the management of the physical aspects related to the city life (e.g., weather monitoring, infrastructure control, and social engagement). Figure 12 is a screenshot from the *Toolsmart* [24] project.

### 7.2. Exploiting the Computing Continuum in a CPS

As a use case to study the distribution of the computation loads involving a cyber physical system, in [22], a scenario composed of several emulated IoT devices was put in place. In this use-case, the proposed system follows the architecture shown in Figure 7 and Figure 8; this was completed with a GUI realized in NodeRED, a graphical flow-based development tool enabling the creation of a pipeline through simple Drag&Drop actions.

**Figure 13:**
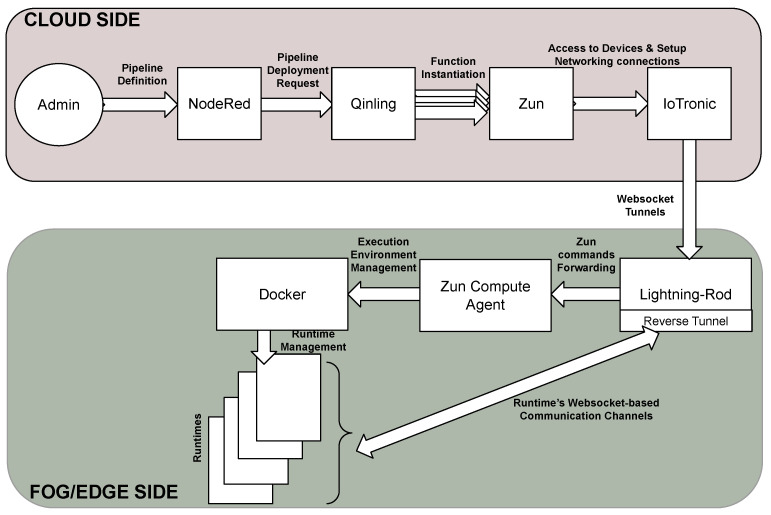
Typical pipeline definition workflow originated by an administrator.

Figure 13 depicts the workflow to create and instantiate the application to manage a CPS.

This example focuses on the creation of an industrial IoT-based sensing system to monitor the temperature of a set of machines. The simplicity of deploying functions on the devices through the exploitation of FaaS facilities is an extremely suitable programming model for such a scenario.

**Figure 14:**
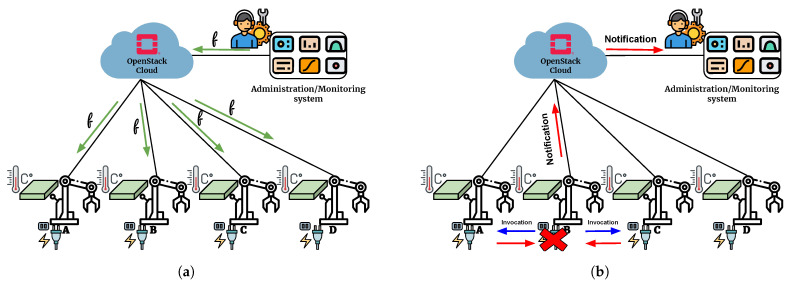
Example of a function-based pipeline on cyber physical system devices through exploitation of a platform offering an edge-based FaaS system: (**a**) The administrator injects the functions generated by pipeline to the devices. (**b**) The workflow is activated when the triggering event occurs.

Through the NodeRED dashboard, the administrator writes the functions that reflect the business logic of the application and easily injects them on the IoT devices (Figure 14a). Our FaaS approach allows the injection of the different software functions into the proper devices (identified through the *nodeSelector*) by exploiting the Qinling component of Openstack.

In the running condition, the temperature is monitored by each device. Each component can autonomously react to external events, such as an overheating event. In this case, a request could be sent to the nearby devices to observe the temperature they detect and conclude if malfunctioning is occurring or if the temperature is actually over the threshold (see Figure 14b). For example, two simple actions to be implemented are to (1) cut the electricity when the machine’s temperature exceeds a certain threshold and (2) send a notification to the monitoring dashboard.

### 7.3. Cooperation among CPSs

To investigate the cooperation occurring among CPSs, we analyzed the possible interactions among vehicles moving in a city and the traffic light subsystem. The smart city is made of several subsystems, each controlling a specific aspect of life (traffic congestion, air pollution, emergency requests, traffic light status, and more). A strong cooperation among such subsystems would enable the vehicles to agree on a path that is able to reduce the air pollution and limit traffic congestion. Each vehicle can be seen as an autonomous CPS that should be put in relation with the surrounding environment managed by the smart city. The cooperation occurs if the traffic management and the traffic light subsystems interact, exchanging info and allowing the actuation of specific actions.

**Figure 15:**
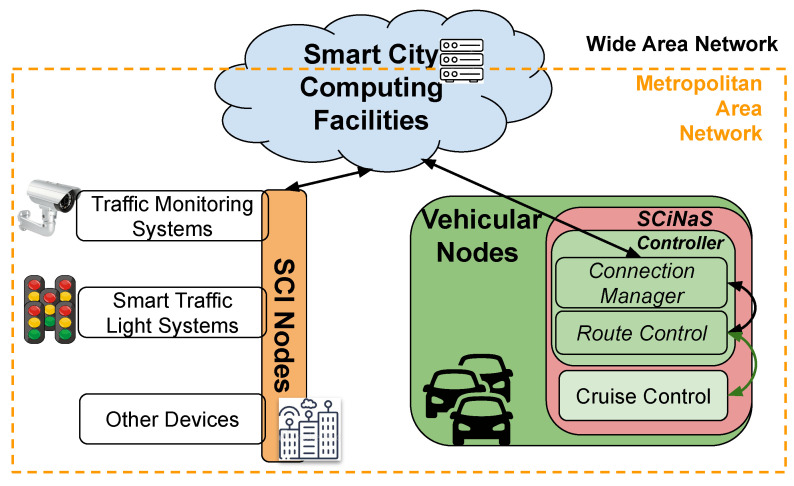
High-level view of cooperation among CPSs in SCiNaS [47].

The proposed architecture exploits the computing continuum approach that was previously described, taking advantage of the interactions among several CPSs. In this way, the computation load can be distributed on the smart city Infrastructure Nodes (SCI Nodes in Figure 15).

In this scenario, cloud computing facilities act as a “*mediator*”/“*data broker*” between SCI Nodes and Vehicular Nodes. Each vehicle receives all the information related to the segments of street it is traveling (the status of the traffic lights, traffic condition, length of the street segment, and max speed allowed) and computes the optimal speed profile to be adopted. This information is sent to a Simulink-based “power-train model” to evaluate and compare the traversing time, fuel consumption, and greenhouse gas emissions against the *New European Driving Cycle* standard [48].

**Figure 16:**
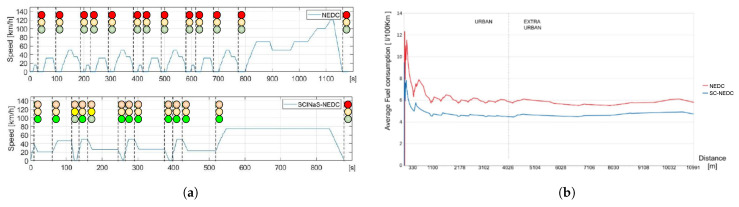
Reduction of traversing time (**a**) and fuel consumption (**b**) when an effective cooperation among city and vehicles is put in place.

As shown in Figure 16a, the cooperation among CPSs reduced the traversing time from 1180 s to less than 900 s. The fuel consumption was also decreased from an average value of 6 to 4.5 L/100 km (see Figure 16b).

## 8. Conclusions

In this paper, we presented some of the main challenges, the state-of-the-art, and solutions to a set of currently unresolved key questions related to CPSs and smart cities. We recognize the great ferment in both the research and development related to cyber physical systems. We highlighted the main lines of research and outlined the possible evolutions and the challenges to be faced.

These challenges include the consideration of interoperability aspects at various levels (protocol and semantic interoperability), the development of distributed IoT applications taking advantage of cloud-native principles, the design and implementation of intelligent orchestration mechanisms injecting automation characteristics in the various parts of the infrastructure, the development of human-centric solutions, and the efficient and secure management of the collected data.

To address these challenges, a holistic approach for the development of IoT based applications for smart cities was detailed considering the application development, orchestration, and infrastructure management parts. The approach is generic and can be applied to manage IoT applications over dynamic and programmable infrastructures, considering the network, computer, and storage. Through a set of intelligent orchestration mechanisms, automation can be injected in the various parts of the infrastructure, while the interplay of resources allocation in the cloud and edge parts of the network can be efficiently managed.

The provided approach was instantiated in a novel CPS framework, the Stack4things platform. Specific considerations were provided regarding the implementation aspects and illustrating the principles and mechanisms offered by the Stack4Things platform. In addition, some application cases were presented that highlighted the usefulness of federating resources to offer services with ever greater added value.

## Figures and Tables

**Table 1:** The mapping of challenges to enabling technologies.

Main Challenge	Sub-Challenge	Enabling Technology
Need for convergence of IoT technologies	IoT Communication Protocols Interoperability	Digital Twins
	IoT Devices Semantic Interoperability	Digital Twins, IoT Semantic Models
	Privacy and Security Aspects	Digital Twins, IoT Cybersecurity mechanisms
IoT applications development and management	Development of distributed and self-adaptive IoT applications	Cloud-native principles, Microservices-based Applications, Containerization
	Software modularity and reusability	Generic IoT functions/enablers
Improve Intelligence and Automation	Dynamic Orchestration Mechanisms	Cloud/Edge Computing Orchestrators
	Automation	Cloud/Edge Computing Orchestrators, Artificial Intelligence, Control Theory
	Massive IoT Deployments	5G, IoT Network Slicing
	Context Awareness	IoT Semantic Models, Distributed Data Management, Distributed AI
Human-centric solutions	Tactile and haptic communications	Design of human-centric interfaces, Tactile Internt technologies
	Involving human-in-the-loop	Internet of Skills (IoS), Augmented Reality/Virtual Reality
Efficient IoT data storage, representation and management	Data management over structured and unstructured data	IoT Semantic Models, IoT data lakes, knowledge graphs
	Distributed data management and analysis	Federated Learning, Distributed AI
	Data Privacy and Security	Blockchain
